# Hydrogen‐Bonding Order Associated With Radical Delocalization in Layered Organic Cathode Materials

**DOI:** 10.1002/anie.2483808

**Published:** 2026-09-16

**Authors:** Alae Eddine Lakraychi, Zeqian Zhang, Oliver Lin, Eric D Walter, Jiaqi Wang, Harshan Reddy Gopidi, Eliot F. Woods, Ruozhu Feng, Wei Wang, Pieremanuele Canepa, Brett A. Helms, Kamila M. Wiaderek, Qian Chen, Yan Yao, Ying Chen

**Affiliations:** ^1^ Energy Storage Research Alliance Argonne National Laboratory Lemont Illinois USA; ^2^ Department of Electrical and Computer Engineering University of Houston Houston Texas USA; ^3^ Texas Center for Superconductivity University of Houston Houston Texas USA; ^4^ Department of Chemistry University of Washington Seattle Washington USA; ^5^ Physical and Computational Sciences Directorate Pacific Northwest National Laboratory Richland Washington USA; ^6^ Department of Chemistry University of Illinois Urbana Illinois USA; ^7^ Materials Research Laboratory University of Illinois Urbana Illinois USA; ^8^ X‐ray Science Division Argonne National Laboratory Lemont Illinois USA; ^9^ Energy and Environment Directorate Pacific Northwest National Laboratory Richland Washington USA; ^10^ The Molecular Foundry Lawrence Berkeley National Laboratory Berkeley California USA; ^11^ Materials Sciences Division Lawrence Berkeley National Laboratory Berkeley California USA; ^12^ Department of Materials Science and Engineering University of Illinois Urbana Illinois USA; ^13^ Department of Earth and Environmental Engineering Columbia University New York New York USA; ^14^ Columbia Climate School Columbia University New York New York USA

**Keywords:** EPR spectroscopy, hydrogen bonds, microcrystal electron diffraction, NMR spectroscopy, organic cathode materials

## Abstract

Organic electrode materials (OEMs) offer a sustainable alternative to inorganic cathodes but typically suffer from low intrinsic electronic conductivity. The material historically reported as bis‐tetraaminobenzoquinone (TAQ) exhibits bulk conductivity two orders of magnitude higher than its close analogue tetraaminophenazine‐tetraone (TAPT), despite their common precursor and nearly identical short‐range structure. This disparity has previously been attributed to distinct molecular skeletons, with TAQ proposed to adopt a piperazine‐type linkage and TAPT a pyrazine ring. Here, combining synchrotron x‐ray scattering, solid‐state ^15^N and ^13^C nuclear magnetic resonance (NMR), microcrystal electron diffraction (MicroED), continuous‐wave and pulsed electron paramagnetic resonance (EPR) spectroscopy, we show that “TAQ” has the same pyrazine‐based molecular structure as TAPT, with no evidence for piperazine core or keto–enol tautomerization. Their key difference lies in synthesis‐dependent degree of crystallinity: “TAQ” exhibits greater long‐range structural coherence and a crystallographically resolved intermolecular hydrogen‐bonding network, whereas TAPT is more disordered. EPR further shows that the higher structural order in “TAQ” is associated with a strongly delocalized π‐spin manifold, while TAPT favors localized or weakly delocalized radicals and stronger local spin coupling. These findings identify degree of crystallinity and hydrogen‐bonding order as key structural features associated with radical delocalization and enhanced conductivity in organic electrodes.

## Graphical abstract

“TAQ” and TAPT share the same pyrazine‐based molecular structure but differ in degree of crystallinity. Combined x‐ray scattering, solid‐state NMR, MicroED, and EPR reveal that “TAQ”'s higher crystallinity and ordered hydrogen‐bonding network are associated with enhanced radical delocalization and conductivity, highlighting structural order as a design principle for organic cathodes.

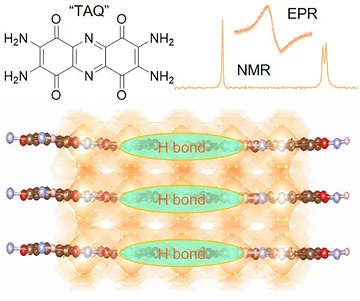

## 1 Introduction

Organic electrode materials (OEMs), composed of earth‐abundant elements such as C, H, N, O, and S, offer a sustainable alternative to the metal‐oxide electrodes that dominate today's lithium‐ion batteries [1, 2, 3, 4, 5]. This compositional advantage also comes with a limitation: most OEMs are electronically insulating (<10^−10^ S cm^−1^) due to fully occupied valence bands and large band gaps [2]. Practical electrodes therefore require large fractions of conductive carbon to achieve adequate utilization, reducing the active material loading and offsetting the energy density advantages that OEMs would otherwise provide [2, 3, 5].

Strategies to impart electronic conductivity to organic solids have been extensively developed in the broad field of organic conductors [6, 7], where the central challenge is to generate mobile charge carriers while establishing efficient intermolecular conduction pathways [8, 9]. Three architectural strategies dominate. Covalent polymerization into conjugated or radical‐bearing backbones, combined with p‐ or n‐type doping, yields conducting polymers with conductivities ranging from semiconducting to metallic (10^−1^–10^3^ S cm^−1^) [10, 11, 12, 13, 14, 15, 16, 17, 18]. Coordination of organic ligands with transition‐metal centers produces conductive metal‐organic frameworks (MOFs) and coordination polymers (CPs), in which π–d hybridization enables charge transport with conductivities of 10^−7^–10^2^ S cm^−1^ [19, 20, 21, 22, 23, 24, 25, 26]. Donor‐acceptor co‐crystallization yields charge‐transfer (CT) complexes, in which partial electron transfer between molecular components generates charge carriers and yields conductivities of 10^−6^–10^1^ S cm^−1^ [27, 28, 29, 30, 31]. Although effective, all three strategies rely on external charge‐generation mechanisms—dopants, coordinated metals, or CT partners—that complicate purification, scaling, and electrochemical interpretation. They also consume a portion of the theoretical capacity and often restrict storage to anions, making it difficult to deploy these materials as electrodes in alkali‐ion cells.

A more attractive route for battery cathodes would be a single‐component, purely organic crystalline solid in which intrinsic carriers arise from the molecular framework itself, without external dopants or coordinated metals [32, 33, 34]. Few such materials are known [35]. Among them, bis‐tetraaminobenzoquinone (TAQ, also referred to as BTABQ or TDT) stands out, with its bulk conductivity of at least 10^−6^ S cm^−1^ [36, 37, 38, 39, 40, 41, 42], approximately two orders of magnitude higher than that of its analogue tetraaminophenazine‐tetraone (TAPT, ∼10^−8^ S cm^−1^) [43], even though both are synthesized from the same precursor (tetraminobenzoquinone, TABQ) by self‐condensation under different conditions. Previous reports attributed this disparity to differences in molecular unit, with TAQ proposed to form a piperazine‐type linkage [36, 40] and TAPT a pyrazine ring. Yet the local atomic structures of the two materials are nearly indistinguishable at short range, and the proposed structural distinction has not been directly tested with hydrogen‐resolved crystallography or solid‐state NMR. The structural origin of this ∼100× conductivity gap therefore remains an open question and offers a particularly clean case study for understanding what controls intrinsic conductivity in single‐component organic redox solids.

In this work, we address this unresolved question by examining the structural and spectroscopic factors associated with the conductivity difference between TAQ and TAPT using complementary synchrotron X‐ray scattering, solid‐state NMR, MicroED, and EPR spectroscopy. We demonstrate that TAQ has the same molecular structure as TAPT, with no evidence for piperazine linkages or keto–enol tautomerization. Thus, TAQ and TAPT are not distinct molecular compounds, their differences arise from materials‐level structural order: TAQ exhibits a higher degree of crystallinity and a more ordered intermolecular hydrogen‐bonding network, which are associated with a more delocalized radical manifold. In contrast, the more disordered TAPT structure favors localized or weakly delocalized radicals with stronger spin coupling. These results indicate that synthesis‐dependent structural order, rather than differences in covalent molecular structure, is associated with radical delocalization and electronic transport in these organic cathode materials.

## 2 Results and Discussion

### 2.1 Distinct Synthesis, Morphology, Crystallinity, and Electronic Conductivity

Self‐condensation of TABQ was conducted under organic and aqueous conditions to synthesize TAQ and TAPT, respectively, following previously reported procedures (Figure 1A) [36, 43]. The resulting powders exhibit distinct morphologies and sizes: TAQ comprises uniform micro‐rods of 2–5 µm, whereas TAPT consists of irregularly shaped particles of 5–10 µm (Figure 1B).

**FIGURE 1:**
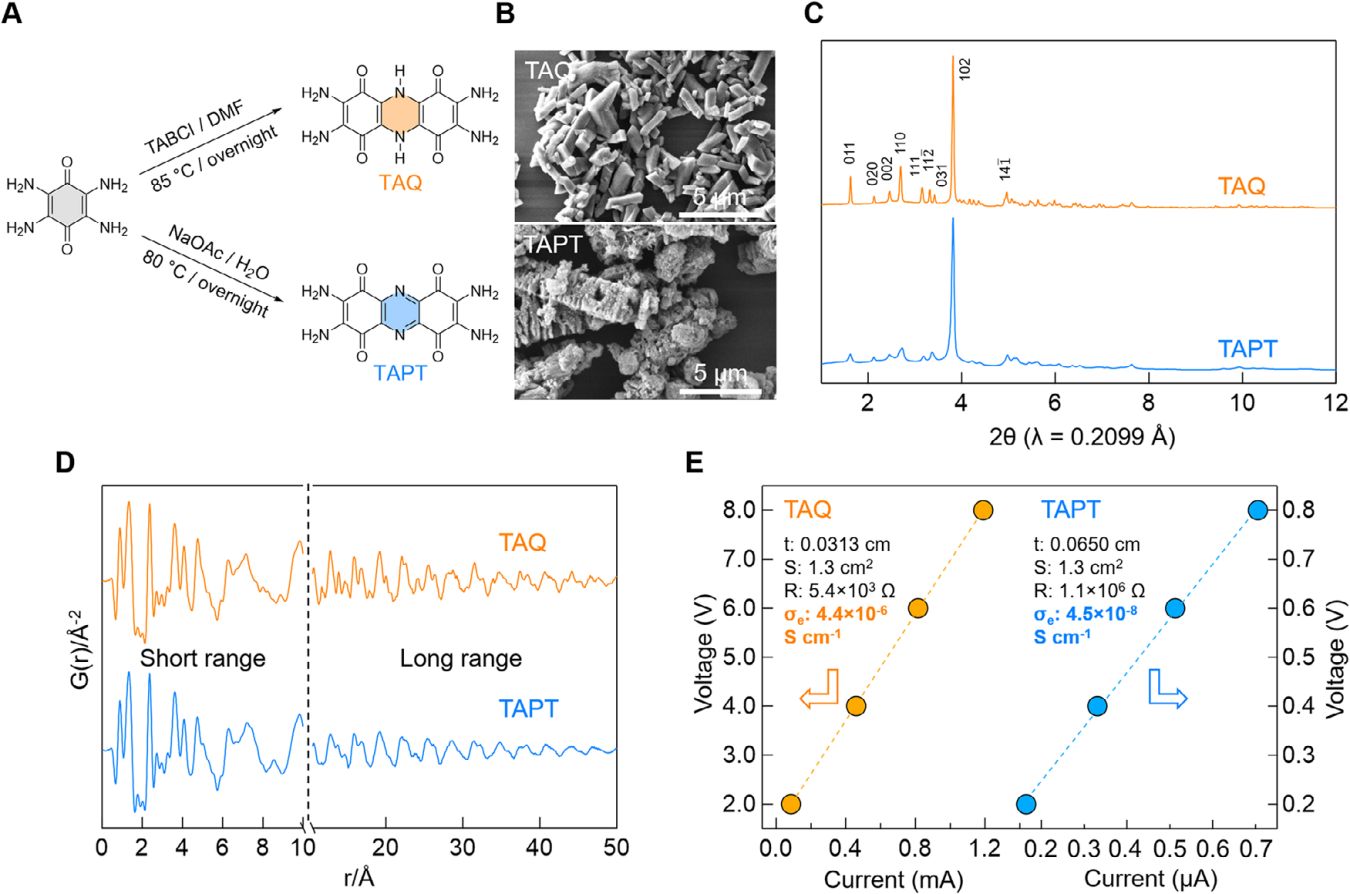
Synthesis, morphology, crystallinity, and electronic conductivity of TAQ versus TAPT. (A) Synthetic routes to TAQ and TAPT via self‐condensation of TABQ under organic and aqueous conditions, respectively. (B) SEM images of TAQ and TAPT powders. (C) Corresponding synchrotron PXRD patterns. (D) Corresponding pair distribution function (PDF) patterns. (E) Room‐temperature electronic conductivity of cold‐pressed pellets of TAQ and TAPT measured in a two‐probe cell configuration.

Synchrotron powder x‐ray diffraction (PXRD) patterns of both compounds display diffraction peaks at identical Bragg positions (Figure 1C), indicating similar underlying crystal structures, in agreement with prior reports [36, 43]. However, TAQ exhibits significantly sharper and more intense reflections, indicative of a higher degree of crystallinity. This observation was consistently reproduced across multiple batches (Figure S1), demonstrating the reproducibility of the difference in structural order. Pair distribution function (PDF) analysis further reveals the distinct degree of structural organization between TAQ and TAPT (Figure 1D). In the long‐range region (10 to 50 Å), TAQ shows well‐defined features, reflecting extended structural coherence, whereas TAPT displays broadened and less distinct features, consistent with reduced long‐range order. This difference likely originates from the distinct crystallization environments provided by the respective solvents during synthesis, where complete dissolution of the precursor in DMF enables a homogeneous reaction environment and more controlled crystal growth, while the limited precursor solubility in water results in a heterogeneous reaction environment that favors rapid precipitation and reduced structural ordering [44]. In contrast, both compounds exhibit nearly identical inter‐atomic distance distributions in the short‐range region (0 to 10 Å), suggesting that their short‐range structures are essentially identical. This interpretation is further supported by Fourier transform infrared (FTIR), as TAQ and TAPT exhibit highly similar spectral features (Figure S2). Diffuse reflectance UV‐Vis‐NIR spectroscopy further reveals similar optical absorption features for TAQ and TAPT (Figure S3), including visible‐region bands and broad near‐infrared absorption associated with their conjugated electronic structures. However, TAQ exhibits sharper absorption features than TAPT, consistent with its higher degree of crystallinity and reduced energetic disorder.

Room‐temperature electronic conductivity measurements performed on cold‐pressed pellets reveal a pronounced difference between the two compounds (Figure 1E). TAQ exhibits a bulk conductivity of 4.4×10^−6^ S cm^−1^, which is two orders of magnitude higher than that of TAPT (4.5×10^−8^ S cm^−1^), in line with the reported conductivity difference [36, 38, 40, 41, 43]. We note that these values represent effective pellet conductivities rather than intrinsic single‐crystal or intraparticle conductivities. As in other polycrystalline organic solids, the bulk conductivity may be influenced by interparticle contacts, grain boundaries, and contact resistance. To minimize these effects, TAQ and TAPT pellets were prepared using the same pellet preparation procedure and electrode contact configuration, and the conductivity difference was reproduced across independently synthesized batches.

### 2.2 Same Molecular Unit, but More Ordered Hydrogen‐Bonding Network for “TAQ”

Since PXRD and PDF analyses are generally not highly sensitive to proton positions, to directly test the previously proposed piperazine versus pyrazine distinction between TAQ and TAPT, we synthesized ^15^N‐labelled analogues of both compounds and performed solid‐state NMR measurements. In these experiments, ^15^N direct polarization (DP) detects nitrogen sites independent of nearby protons, whereas ^1^H–^15^N cross‐polarization (CP) selectively enhances nitrogen atoms close to protons and is therefore diagnostic of N─H environments.

The ^15^N NMR spectra show that TAQ and TAPT contain the same nitrogen‐containing molecular unit, with no evidence for protonated central N sites in TAQ. In the ^15^N direct‐polarization spectrum of TAPT, two resonances appear at 70 and 307 ppm with an integration ratio of 2:1, corresponding to the four amino N atoms and two pyrazinic N atoms, respectively (Figures 2A and S4). In contrast, the ^1^H–^15^N CP spectrum acquired with a short contact time of 200 µs shows only the 70 ppm amine resonance, consistent with the absence of directly bonded protons on the central N atoms. TAQ shows the same behavior: its ^1^H–^15^N CP spectrum also contains only the amino N signal near 70 ppm (Figure 2B), rather than the additional CP signal expected for protonated central N sites. No ^1^H–^15^N CP signal is observed between 100 and 1200 ppm, even with a long contact time of 3000 µs (Figure S5). This absence rules out both piperazine linkage and keto–enol tautomerization that would generate ─C═NH groups, which should produce ^1^H–^15^N CP signals in the 200–350 ppm region. The ^1^H–^15^N Heteronuclear Correlation (HETCOR) spectra further support this assignment: both TAPT and TAQ show strong cross‐peaks between the ^1^H resonance centered at 6 ppm and the ^15^N resonance at 70 ppm, confirming directly bonded amine N─H groups while showing no evidence for other protonated N sites (Figure 2C,D). Therefore, TAQ and TAPT share the same pyrazine‐based molecular structure, suggesting that they are chemically identical at the molecular level. For clarity and consistency with the existing literature, we refer to the material obtained through the DMF‐based synthesis as “TAQ” to distinguish it from the material obtained through water‐based synthesis TAPT. The quotation marks indicate that “TAQ” denotes the historically reported synthesis‐derived material, rather than a distinct TAQ molecular structure.

**FIGURE 2:**
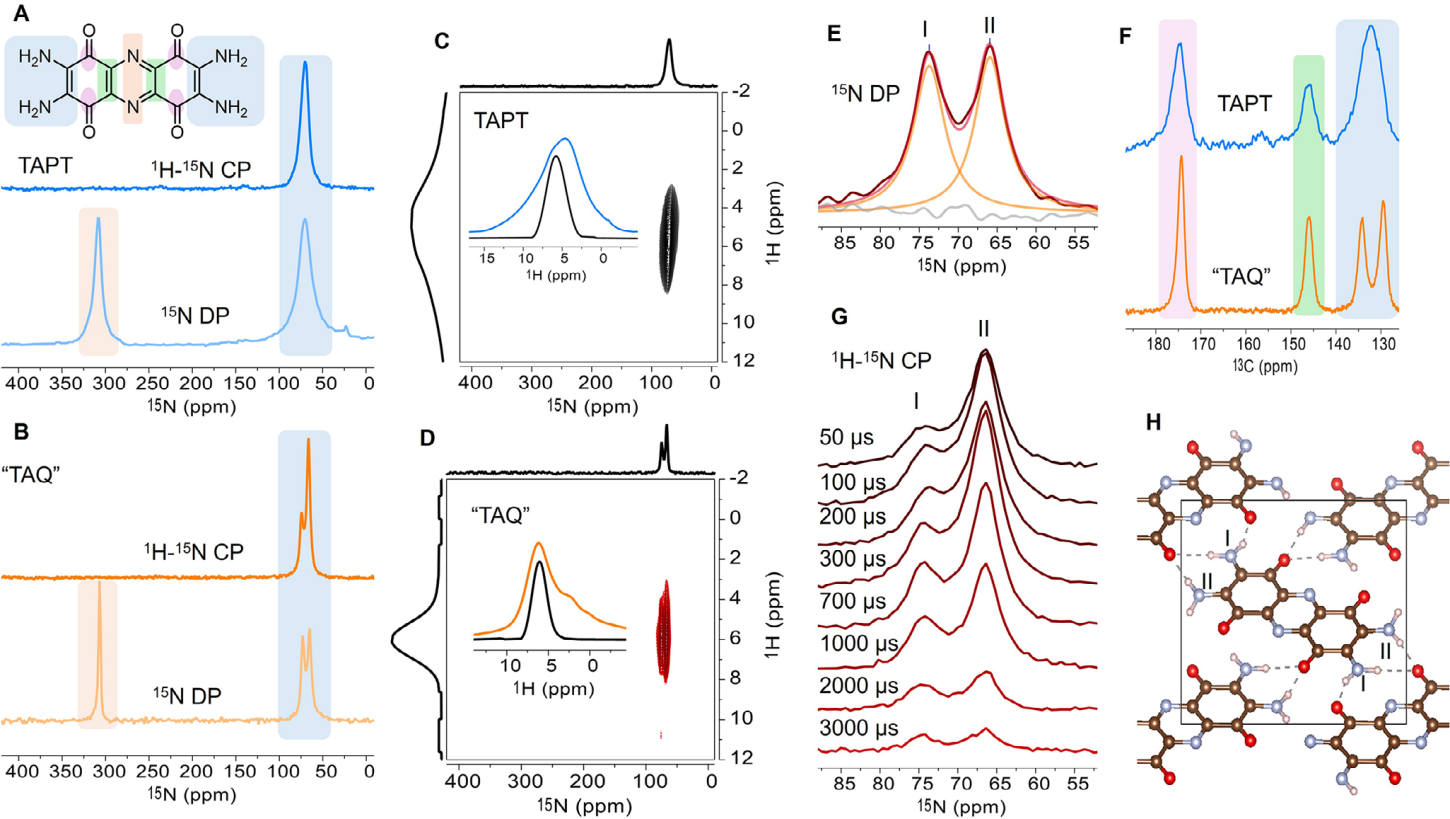
Solid‐state NMR shows that “TAQ” and TAPT share the same molecular building unit, while “TAQ” has a more ordered hydrogen‐bonding network. (A, B) Comparison of ^1^H–^15^N CP and ^15^N DP NMR spectra for (A) TAPT and (B) “TAQ”, acquired with a CP contact time of 200 µs. (C, D) ^1^H–^15^N HETCOR spectra for (C) TAPT and (D) “TAQ”, acquired with a contact time of 200 µs. (E) Deconvolution of the resolved amino ^15^N doublet in “TAQ”. (F) Comparison of ^1^H–^13^C CP spectra of “TAQ” and TAPT. (G) ^1^H–^15^N CP spectra of the amino ^15^N resonance in “TAQ” acquired at varying CP contact times. (H) Schematic illustration of two distinct amine‐based hydrogen‐bonding environments in “TAQ”, arising from in‐plane orientational order.

Although “TAQ” and TAPT share the same molecular unit, their ^15^N spectra reveal substantially different distributions and dynamics of the local nitrogen environments. Three spectral differences support this conclusion. First, “TAQ” exhibits much narrower ^15^N linewidths than TAPT: the pyrazinic and amino N linewidths are approximately 200 and 300 Hz in “TAQ”, compared with approximately 600 and 1100 Hz in TAPT, respectively. Second, the ^15^N spin–lattice relaxation time, *T*_1_, of the pyrazinic nitrogen increases from approximately 12 s in TAPT to 60 s in “TAQ” (Figure S6), indicating substantially less efficient longitudinal relaxation in “TAQ”. This longer *T*_1_ is consistent with a more rigid local environment, although differences in paramagnetic relaxation may also contribute. Quantitative EPR measurements presented below show that TAPT contains approximately twice the radical concentration of “TAQ”, however, under a simple model assuming equivalent paramagnetic relaxitivity in the two materials, this twofold difference in radical concentration cannot account for the approximately fivefold difference in ^15^N relaxation rate. Third, the amino N resonance appears as a resolved approximately 1:1 doublet in “TAQ” (Figure 2E), indicating two distinct but well‐defined local environments. In contrast, TAPT exhibits a broad, unresolved resonance centered near the middle of the “TAQ” doublet, consistent with a broader distribution of local structures. Together with the XRD results in Figure 1C, these NMR features demonstrate that “TAQ” has more homogeneous molecular packing and more highly ordered local structure than TAPT.

The ^13^C NMR spectra independently support the higher structural order of “TAQ” and are consistent with the absence of keto–enol tautomerization. The ^1^H–^13^C CP spectrum of “TAQ” exhibits narrower linewidths than that of TAPT, and the broad singlet at 132 ppm in TAPT resolves into a doublet in “TAQ” (Figure 2F). The resonance at 145 ppm was previously assigned to a –C═NH species and interpreted as evidence for keto–enol tautomerization [36]. However, comparison of the ^1^H–^13^C CP buildup and efficiency data supports an alternative assignment to the four carbon atoms directly bonded to the pyrazinic N atoms (Figure S7). This resonance exhibits the lowest CP efficiency, indicating the weakest through‐space polarization transfer from nearby protons and therefore the largest average separation from proton‐rich environments. This assignment is also consistent with the ^1^H–^15^N CP spectra, which show no evidence for protonated imine‐like nitrogen environments.

A uniform offset between our ^13^C chemical shifts and previously reported “TAQ” values is best explained by different chemical‐shift referencing, not by a different molecular unit. The reported ^13^C chemical shifts of TAPT closely match our measured values [43], whereas the reported ^13^C shifts of “TAQ” are systematically 4.8–5.1 ppm lower than ours for all resonances (Table S1) [36]. Because this offset is nearly constant across the spectrum, it likely reflects a referencing difference. If “TAQ” contained a piperazine linkage rather than the pyrazine ring identified here, the change in ring oxidation state would be expected to alter the relative ^13^C chemical shifts (Table S1), rather than simply shift the entire spectrum by an approximately constant amount. Therefore, after accounting for the referencing offset, the close correspondence of the ^13^C spectral features further supports that the “TAQ” examined in this study is structurally equivalent to previously reported “TAQ”.

The resolved amine doublet in “TAQ” indicates two distinct, ordered hydrogen‐bonding environments within the crystal structure. To determine the origin of this doublet, we acquired ^1^H–^15^N CP spectra of “TAQ” with contact times ranging from 50 to 3000 µs (Figure 2G). The 66 ppm component, denoted N‐II, shows faster CP buildup than the 74 ppm component, denoted N‐I. Because faster CP buildup generally indicates more efficient polarization transfer from nearby protons, this result suggests that N‐I has a longer effective H–N distance or weaker local ^1^H–^15^N coupling. Since the four amine groups are symmetry‐equivalent within an isolated molecular unit, the two ^15^N environments must arise from intermolecular interactions rather than from different covalent structures. The crystal structure of “TAQ” provides a natural explanation (Figure 2H): two amino groups on the same side of the molecule experience different hydrogen‐bonding environments, with one participating in two N─H···O hydrogen bonds and the other participating in only one. Previous studies have shown that stronger participation of an amine group as a proton donor in N─H···O hydrogen bonding can shift its ^15^N resonance downfield [45, 46]. We therefore assign the 1:1 doublet to two amino N sites with different hydrogen‐bonding strengths. The downfield N‐I site engages in stronger intermolecular hydrogen‐bonding, which lengthens the N─H bond, slows CP buildup, and shifts the ^15^N resonance to higher frequency. In contrast, TAPT shows a broad, unresolved amino ^15^N resonance centered near the middle of 1:1 doublet observed in “TAQ”. This does not exclude the presence of local hydrogen bonding in TAPT; rather, it indicates that the amino‐based hydrogen‐bonding geometries in TAPT are more heterogeneously distributed and are not organized into the same long‐range ordered hydrogen‐bonding network resolved for “TAQ”.

MicroED on “TAQ” produced a hydrogen‐atom‐resolved crystal structure that directly confirms the pyrazine assignment from NMR. High‐resolution data collection under cryogenic conditions, coupled with high crystallinity of our samples, allowed determination of either presence or absence of hydrogen atoms to visualize the hydrogen‐bonding network with kinematical structure refinement. The diffraction pattern exhibits reflections extending beyond the detector edge at 0.58 Å (Figure 3A). The crystal structure was solved by intrinsic phasing using ShelxT in the monoclinic space group P2_1_/c, with unit cell parameters a = 4.8936(5) Å, b = 11.285(1) Å, c = 9.841(1) Å, β = 102.097(9)°, and V = 531.37(10) Å^3^, in agreement with literature values [36, 38]. Refinement proceeded smoothly without significant constraints (see details in SI), and the final structure is presented in Figures 3B and S8. Corresponding bond lengths are shown in Figure 3C and Tables S2–S6.

**FIGURE 3:**
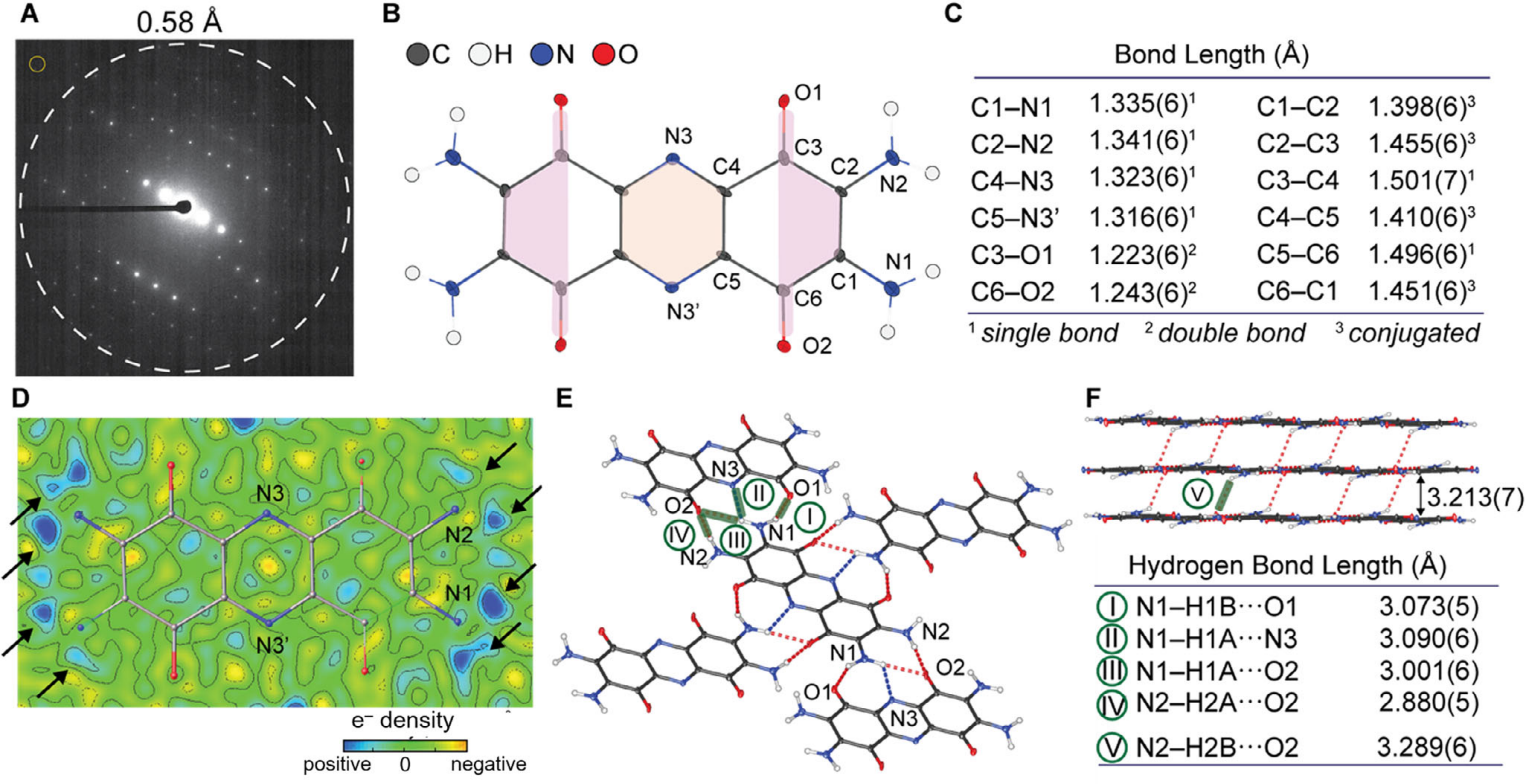
MicroED crystal structure of “TAQ” reveals a pyrazine core and an ordered hydrogen‐bonding network. (A) Electron diffraction pattern from a “TAQ” particle, showing high crystallinity and diffraction spots up to 0.58 Å, indicated by the yellow circle extending beyond detector edge. (B) MicroED crystal structure of “TAQ”, with displacement ellipsoids drawn at the 50% probability level and atoms labelled in the asymmetric unit. Compartmentalized electron conjugation is represented with pyrazine (orange) and peripheral quinone unit (pink). (C) Table includes bond lengths in Å, noted with the bond order. (D) Difference Fourier map of the structural model without hydrogen assignment, with arrows indicating the location of hydrogen atoms. (E, F) Intermolecular hydrogen‐bonding network, with in‐plane (E) and out‐of‐plane (F) hydrogen bonds highlighted with green circles. Corresponding hydrogen bond lengths are indicated in the inset chart.

Bond length analysis reveals that “TAQ” crystallizes in the quinonoid form with a pyrazine core, as evidenced by C─O bond lengths (C3–O1 = 1.223(6) Å and C6–O2 = 1.243(6) Å) and C─N bond lengths (C4–N3 = 1.323(6) Å and C5–N3’ = 1.316(6) Å), consistent with carbonyl (C═O) character and conjugated C═N bonds arising from the pyrazine ring, respectively. Moving toward the molecular extremities, the amino C─N bonds (C1–N1 = 1.355(6) Å, C2–N2 = 1.341(6) Å) are intermediate between typical single (∼1.43 Å) and double (∼1.24 Å) bonds, consistent with partial single‐double or conjugated character. Despite overall molecular planarity, π‐electron delocalization is confined within the quinone units and pyrazine core but is interrupted between these units, as illustrated in Figure 3B.

Hydrogen atoms were determined experimentally from the difference Fourier map (Figure 3D). Four distinct maxima of positive electron density are observed near the amino nitrogen atoms (N1 and N2). In contrast, no electron density attributable to hydrogen is detected near the pyrazinic N atoms (N3), further confirming the pyrazine core. Weaker electron density features in the top‐right and bottom‐left regions of the map, related by inversion symmetry, are hydrogen atoms displaced out of the molecular plane, as discussed below.

The experimentally resolved hydrogen positions were refined freely and directly enable identification of the hydrogen‐bonding network within the “TAQ” crystal. Following the default hydrogen‐bond geometry criteria in SHELX (namely an H···A distance below r(A) + 2.000 Å and a D–H···A angle greater than 110°, where D, H, and A denote the donor, hydrogen, and acceptor atoms and r(A) is the van der Waals radius of the acceptor), five hydrogen bonds were identified: four in‐plane and one out‐of‐plane, as illustrated in Figure 3E,F, respectively. The corresponding bond lengths are summarized in the chart. The amino N atoms act as hydrogen bond donors, interacting with three carbonyl O and one pyrazinic N from neighboring molecules (Figure 3E). Specifically, H1A on N1 forms a hydrogen bond I with carbonyl O1, while H1B engages in bifurcated hydrogen bonding II and III with both pyrazinic N3 and carbonyl O2. H2A shares a hydrogen bond IV with N2. The remaining hydrogen bond V is formed between H2B, which is slightly tilted out of the molecular plane, and an intermolecular carbonyl O2 in the lower plane (Figure 3F). Visualized by the determination of hydrogen atoms, the interlayer interaction is stronger than van der Waals interaction and π‐π stacking interactions alone.

This ordered hydrogen‐bonding arrangement provides a structural basis for the two amine environments observed by ^15^N NMR. In the MicroED structure, amino groups that are covalently equivalent within an isolated “TAQ” molecule become distinguishable in the crystal because they participate in different intermolecular hydrogen‐bonding. Some amino hydrogen atoms are involved in stronger or bifurcated in‐plane hydrogen‐bonding, whereas the other hydrogen atom participates in a weaker out‐of‐plane interaction. This crystallographically resolved asymmetry explains why the amino ^15^N resonance of “TAQ” splits into a 1:1 doublet, while the less ordered packing in TAPT produces a broad unresolved amine signal.

### 2.3 Crystallinity‐Dependent Paramagnetic Redox Species

Having established that “TAQ” and TAPT share the same molecular unit but differ in long‐range structural order, we now ask why these molecularly similar materials differ by two orders of magnitude in bulk conductivity. EPR is a particularly informative probe here because it directly measures the unpaired‐electron carriers responsible for transport in radical‐rich organic solids. Here we used CW and pulsed EPR to determine how crystallinity and hydrogen‐bonding order affect the electronic spin species in these materials. CW EPR detects unpaired electrons, while the line shape, linewidth, g tensor, and temperature‐dependent intensity provide information about whether these spins are localized on individual molecules, weakly delocalized over several molecules, or part of a more delocalized conduction‐like spin manifold [47, 48]. In this regard, broad and asymmetric Dysonian‐like signals are commonly associated with strongly delocalized spins, whereas sharp Lorentzian‐like signals are more characteristic of localized or weakly delocalized organic radicals [47, 49, 50, 51].

Room‐temperature CW EPR indicates that “TAQ” is dominated by a broad delocalized spin component, whereas TAPT contains a sharp weakly delocalized and a broader component. As reported previously, “TAQ” exhibits a broad asymmetric EPR signal, which has been described using a modified Dysonian line shape. We observe a similar broad signal for “TAQ”, while TAPT displays a much narrower signal with a more conventional rhombic powder pattern (Figure 4A,B). Although organic radicals are often approximated using an isotropic g value, the radicals in “TAQ” and TAPT reside in ordered or partially ordered polycrystalline solids; therefore, g anisotropy can contribute to the powder EPR spectra because molecular motion is restricted. We therefore fitted the spectra using EasySpin with one or two spin systems and included the microwave phase parameter, mwPhase, as a phenomenological measure of absorption/dispersion admixture, where 0 corresponds to pure absorption and π/2 to pure dispersion. Detailed fitting procedures are provided in Supporting Information.

**FIGURE 4:**
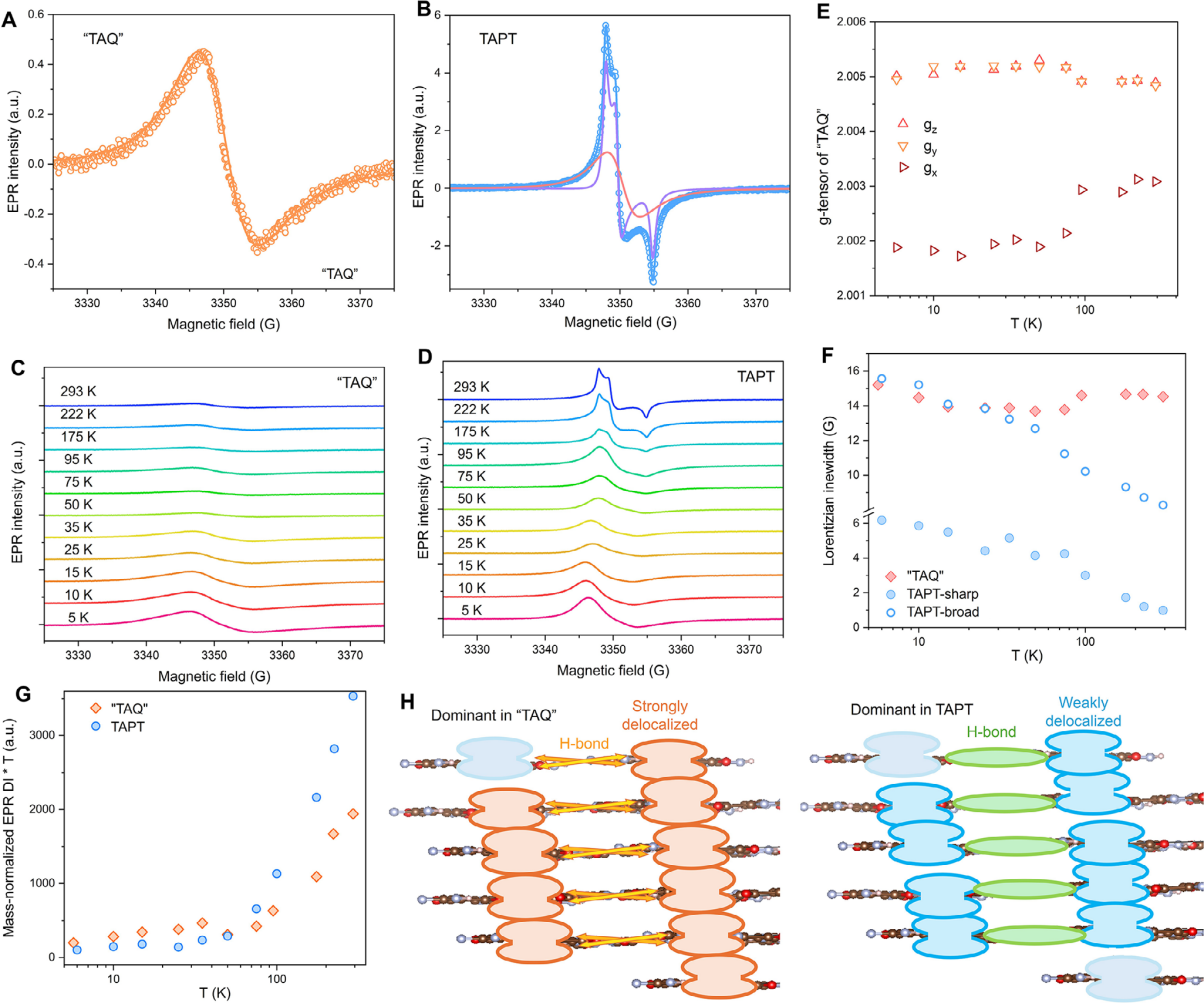
Temperature‐dependent CW EPR reveals that long‐range structural coherence controls the balance between delocalized and localized radical species. (A, B) Room‐temperature CW EPR spectra and corresponding simulations for (A) “TAQ” and (B) TAPT. “TAQ” is dominated by a broad Dysonian‐like component, whereas TAPT requires both a sharp weakly delocalized component and a broader component. (C, D) Temperature‐dependent CW EPR spectra of (C) “TAQ” and (D) TAPT. Temperature dependence of (E) the fitted g values for “TAQ”, (F) Lorentzian linewidths for “TAQ” and TAPT, and (G) mass‐normalized DI × T, where DI is the double‐integrated EPR intensity. DI × T is approximately constant for Curie‐like localized spins, whereas deviations indicate changes in spin delocalization, coupling, or EPR visibility. (H) Schematic illustration of radical species in “TAQ” and TAPT, highlighting the predominance of strongly delocalized spins in “TAQ” due to long‐range structural coherence and the prevalence of localized or weakly delocalized spins in TAPT due to reduced structural order.

The fitted EPR parameters support a single dominant spin environment in “TAQ” but two coexisting spin environments in TAPT. The “TAQ” spectrum is satisfactorily fitted with one spin system showing axial g anisotropy, with g_z_ ≈ g_y_ = 2.0048, g_x_ = 2.0031, a Lorentzian linewidth of 14.6 G, and mwPhase = 0.29 (Figure 4A). In contrast, TAPT requires two components for satisfactory fitting (Figure 4B): a sharp component with g_z_ = 2.0058, g_y_ = 2.0048, g_x_ = 2.0017, and a linewidth of 1.0 G; and a broader component with g_iso_ = 2.0045, a linewidth of 8.3 G, and mwPhase = 0.19. Interestingly, TAPT*, a less crystalline form of TAPT with similar interlayer distance (Figure S9), displays an EPR spectrum qualitatively similar to that of TAPT and still requires two components for satisfactory fitting, a result further supported by Q‐band EPR (Figure S10). However, relative to TAPT, the sharp component in TAPT* narrows from 1.0 to 0.8 G, the mwPhase of the broad component decreases from 0.19 to 0.02, and the fraction of the sharp component increases from approximately 30% to 60%. These trends indicate that multiple linewidth‐narrowing and linewidth‐broadening mechanisms coexist. The broad component in TAPT/TAPT*, especially when the phase contribution is small, is assigned to more localized radicals at defects or structurally less connected regions, where unresolved hyperfine interactions, g‐strain, and heterogeneous local environments are less effectively averaged. The sharp component is assigned to radicals in more structurally similar local environments that are weakly delocalized or dynamically averaged among neighboring TAPT units, likely along the π‐stacking direction, leading to partial averaging of hyperfine and local g‐tensor variations. In contrast, the broad “TAQ” resonance is not interpreted as a simple hyperfine‐broadened signal from isolated localized radicals. Rather, its larger linewidth, stronger absorption/dispersion admixture, higher conductivity, and more ordered crystalline/hydrogen‐bonded framework suggest that additional broadening mechanisms dominate in “TAQ”. In particular, the larger mwPhase supports a more spatially extended spin response within ordered “TAQ” domains that gives rise to a weak Dysonian‐like EPR lineshape. The broad linewidth may also contain contributions from stronger intermolecular spin‐spin interactions enabled by ordered hydrogen‐bonding network. Note here “Dysonian‐like” refers only to the observed absorption/dispersion admixture and does not imply classical metallic conduction‐electron spin resonance. The measured bulk conductivity of “TAQ”, σ∼10^−6^ S cm^−1^, gives an estimated X‐band skin depth of approximately 0.5 m, far exceeding the 313 µm pellet thickness. Thus, the observed asymmetry cannot be attributed to classical bulk Dysonian conduction‐electron spin resonance based on the measured bulk conductivity. Instead, the asymmetry suggests a local microwave response within structurally ordered “TAQ” domains that is stronger than expected from the macroscopic bulk conductivity, reflecting strongly delocalized radicals and possibly higher intradomain electronic connectivity in “TAQ”.

Temperature‐dependent EPR further shows that the delocalized spin manifold in “TAQ” is more robust than that in TAPT. Quantitative EPR spectra were collected from 293 to 5 K with particular care to avoid microwave power saturation, especially at low temperature (Figure 4C,D; Figures S11 and S12). For “TAQ”, the overall line shape changes only modestly with temperature. The principal g values remain nearly constant, with g_z_ ≈ g_y_ staying between 2.0049 and 2.0052 and g_x_ decreasing only slightly from 2.0031 to 2.0020 between 100 and 75 K (Figure 4E). The linewidth also remains nearly unchanged, varying only from 13.9 to 15.2 G (Figure 4F). By contrast, mwPhase increases monotonically from 0.28 at 293 K to 0.56 at 5 K, indicating an increasing dispersion or Dysonian‐like contribution upon cooling (Figure S13).

The temperature dependence of the “TAQ” EPR intensity is consistent with a dominant delocalized spin contribution plus a smaller localized spin population that is most visible at low temperature. The double‐integrated (DI) EPR intensity is proportional to the EPR‐visible magnetic susceptibility. For “TAQ”, DI remains approximately constant from 293 to 50 K (Figure S14), a behavior consistent with a substantial contribution from strongly delocalized charge carriers. Below 50 K, DI increases upon cooling, while DI × T becomes nearly temperature‐independent (Figure 4G). Since Curie‐like localized S = 1/2 spins typically give temperature‐independent DI × T, this low‐temperature behavior indicates that a residual population of localized or weakly interacting spins becomes more apparent below 50 K.

In contrast, TAPT undergoes a much stronger temperature‐dependent change in spin character. Upon cooling from 293 to 5 K, the linewidth of the sharp component increases from 1.0 to 6.2 G, while the linewidth of the broad component increases from 8.3 to 15.6 G (Figure 4F). The g tensor of the sharp component remains nearly constant, but the g value of the broad component changes substantially: it remains near 2.0042 between 293 and 100 K, then decreases to 2.0020 at 75 K and further to 2.0006 at 5 K (Figure S15). At the same time, mwPhase fluctuates between 0.19 and 0.55, and the fitted fraction of the sharp component increases from approximately 30% at 293 K to approximately 75% at 5 K. Because the TAPT spectra contain two overlapping components whose linewidths, fractions, and mwPhase contributions all vary with temperature, these fitted parameters are partially correlated, especially at low temperature where the sharp component broadens substantially. Therefore, the exact numerical values should not be overinterpreted. Nevertheless, the contrast with “TAQ” is clear: “TAQ” maintains a nearly constant linewidth and only minor g‐value changes, whereas TAPT shows pronounced broadening and a strong temperature‐dependent shift of the g values.

The stronger temperature dependence of TAPT suggests that structural disorder promotes carrier trapping, spin localization, or spin‐pairing at low temperatures. DI × T for TAPT decreases much faster than for “TAQ” between 293 and 100 K, then becomes nearly temperature‐independent from 75 to 5 K (Figure 4G). This behavior is consistent with cooling suppressing motional averaging and trapping carriers within a more disordered energy landscape. As the localized or weakly delocalized radicals become confined to fewer molecular units, they become more sensitive to local hyperfine couplings and structural inhomogeneity, producing broader EPR lines at low temperatures. Thus, although TAPT exhibits a room‐temperature radical concentration twice higher than that of “TAQ” (Figure S14), its more rapid decrease in DI × T upon cooling indicates that a substantial fraction of these additional EPR‐visible spins progressively become EPR‐silent at low temperatures, consistent with antiferromagnetic coupling, singlet formation, and/or other local spin‐pairing processes in the more disordered TAPT lattice.

Overall, EPR reveals that crystallinity and hydrogen‐bonding order are strongly associated with the distribution of radical environments in “TAQ” and TAPT. As summarized schematically in Figure 4H, “TAQ” exhibits a broad Dysonian‐like EPR signal consistent with a more spatially extended π‐spin response within the ordered “TAQ” framework that remains comparatively robust upon cooling. TAPT and TAPT* show progressively sharper and less Dysonian‐like spectra as long‐range structural order decreases, indicating an increasing fraction of localized or weakly delocalized radicals. Thus, the common π‐π stacking distance of these materials is not sufficient to define their radical character; instead, long‐range crystallinity and hydrogen‐bonding network determine the balance among strongly delocalized, weakly delocalized or more localized radical species. While quantitative models exist for interpreting temperature‐dependent EPR intensities and linewidths, their application requires simplifying assumptions about carrier identity and the dominant broadening mechanism. However, because multiple radical populations and linewidth mechanisms coexist in “TAQ”/TAPT, the present data do not permit a unique quantitative extraction of spin fractions, delocalization lengths, or carrier mobilities.

While the CW EPR analysis captures the dominant radical species and their correlation with crystallinity, it is less sensitive to minor spin populations at low temperatures and local hyperfine environments. We therefore turned to low‐temperature half‐field and pulsed EPR measurements to probe weakly populated S ≥ 1 states and more localized or weakly delocalized radical species that are not fully resolved in the CW spectra. Half‐field spectra acquired in both perpendicular and parallel detection modes show a weak but distinct signal at the half‐field position, corresponding to the formally forbidden Δm_s_ = 2 transition, for “TAQ” from 5 to 35 K and for TAPT from 5 to 75 K (Figure 5A,B). Such a transition is characteristic of coupled spin systems with total electron spin S ≥ 1, most commonly triplet‐like states [47, 52]. In both materials, the half‐field intensity decreases as temperature increases, consistent with a low‐lying or ground‐state triplet‐like population. However, this temperature dependence alone is not sufficient to unambiguously establish that the triplet is the ground state. Because strongly delocalized, localized, and spin‐coupled species coexist in both “TAQ” and TAPT, the EPR intensity cannot be assigned to a single isolated two‐spin manifold; therefore, a quantitative singlet–triplet energy‐gap analysis is not warranted here.

**FIGURE 5:**
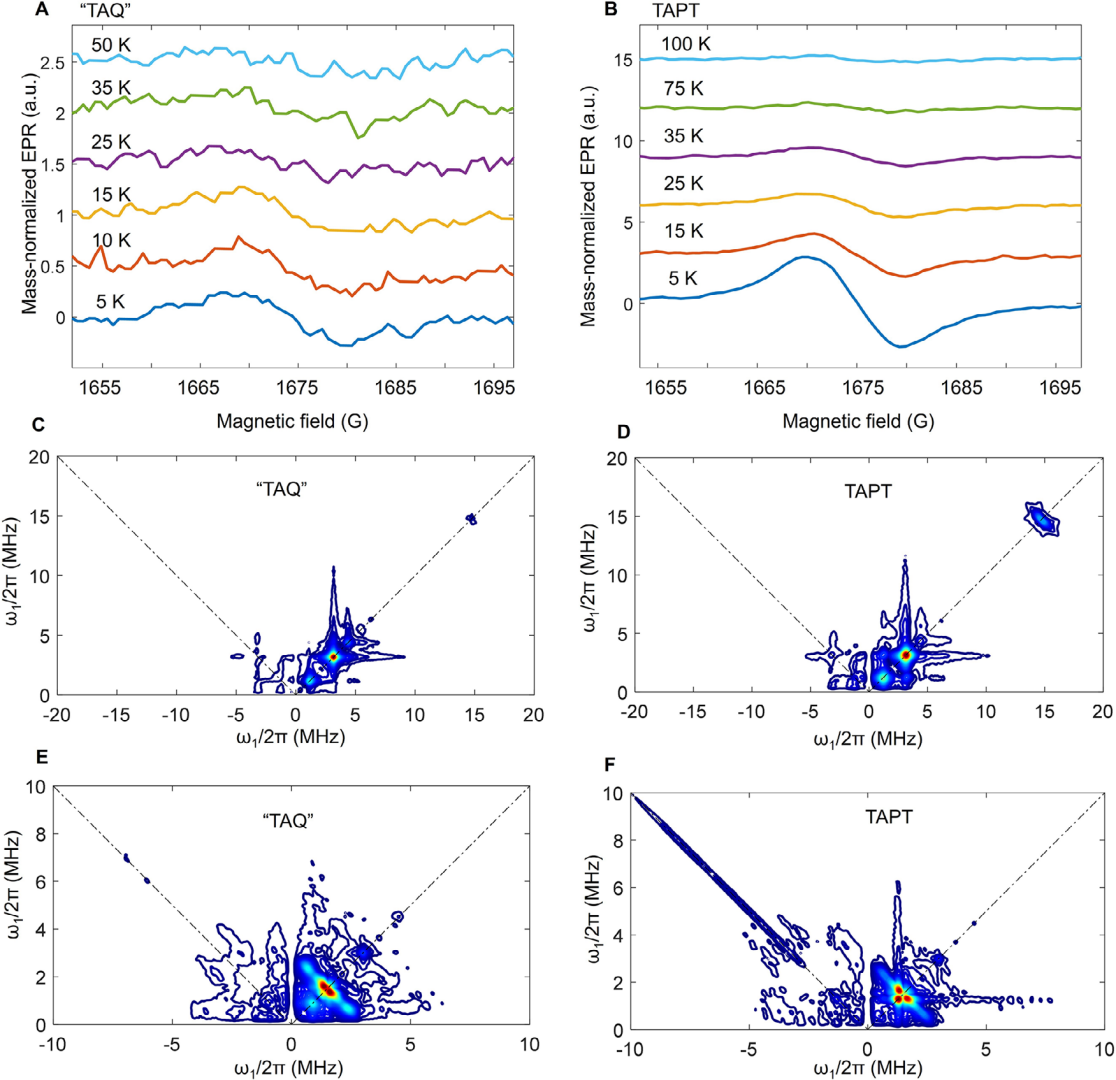
Low‐temperature half‐field and pulsed EPR measurements reveal minor S ≥ 1 and weakly delocalized π‐radicals. (A, B) Half‐field EPR spectra of (A) “TAQ” and (B) TAPT at varying temperatures. (C, D) HYSCORE spectra of natural‐abundance (C) “TAQ” and (D) TAPT at 5 K. (E, F) HYSCORE spectra of ^15^N‐enriched (E) “TAQ” and (F) TAPT at 5 K. The HYSCORE spectra shown here were summed over multiple interpulse delays (Figure S15) to minimize blind‐spot effects.

The much stronger half‐field signal in TAPT indicates that local spin coupling is favored in the less ordered framework. In “TAQ”, the half‐field signal is extremely weak relative to the main resonance, consistent with only a small population of residual spin dimers or clusters. In contrast, the mass‐normalized half‐field intensity of TAPT is approximately an order of magnitude larger than that of “TAQ” and remains observable to substantially higher temperature. This behavior agrees with the temperature‐dependent CW EPR analysis: “TAQ” favors a more delocalized π‐radical manifold, whereas TAPT more readily supports local spin coupling between nearby radicals.

Pulsed EPR was then used to selectively examine the subset of radicals that retain measurable spin coherence at low temperatures. Unlike CW EPR, which detects the total EPR‐visible spin ensemble, pulsed EPR detects only spins with sufficiently long coherence to generate an electron spin echo. Two‐pulse electron spin echo envelope modulation (2P ESEEM) measurements at 5 K gave comparable phase‐memory times (*T*_m_) for “TAQ” and TAPT, with *T*_m_ values of 0.12 µs and 0.15 µs, respectively (Figure S16). These short but measurable *T*_m_ values are sufficient for Hyperfine Sublevel Correlation Spectroscopy (HYSCORE), although they remain shorter than the long coherence times reported for highly optimized organic spin systems [53].

Natural‐abundance HYSCORE spectra show that the echo‐detectable radicals in “TAQ” and TAPT experience similar nitrogen and proton hyperfine environments. At 5 K, the HYSCORE spectra of natural‐abundance “TAQ” and TAPT are very similar (Figures 5C,D and S17). Both spectra show prominent ^14^N features in the (−,+) quadrant, as well as strong cross‐peaks near 1.1, 2.2, and 4.4 MHz in the (+,+) quadrant. A diagonal signal near 14.1 MHz is assigned to weakly coupled ^1^H nuclei. The similarity of the “TAQ” and TAPT HYSCORE patterns indicates that the echo‐detectable spins in both materials couple to comparable local nuclear environments, despite the stronger contribution of delocalized spins in the CW EPR spectrum of “TAQ”.

The weak‐to‐moderate nitrogen couplings suggest that these echo‐detectable radicals are not confined to a single molecular unit. The ^14^N HYSCORE features (Figure 5C,D) resemble the simulated spectra reported for trimer and tetramer porphyrin nanoribbons, in which cationic polarons are delocalized over three to four porphyrin units, corresponding to a coherence length of approximately 2–3 nm [53]. In those systems, distributed point‐dipole and density‐functional theory (DFT)‐based simulations reproduced weak‐to‐moderate ^14^N hyperfine couplings of a few MHz, with representative tensors such as [0.24, 0.16, 5.47] MHz, quadrupolar coupling constants of approximately 2.4 MHz, and asymmetry parameters of 0.6–0.8. This comparison suggests that the echo‐detectable spins in “TAQ” and TAPT are not strictly confined to a single molecular unit with large spin density located at the central pyrazinic N sites. Instead, the HYSCORE spectra are more consistent with weakly delocalized spins that remain distributed over more than one molecular unit through intermolecular electronic coupling along the π‐stacking direction. Unlike the fused porphyrin nanoribbons, however, the amino nitrogens in “TAQ” and TAPT are not directly embedded in the main π‐conjugated pathway but can still contribute to the HYSCORE response, making a quantitative extraction of delocalization length more challenging in the present system.

The ^15^N‐enriched HYSCORE spectra further support weak nitrogen hyperfine coupling, likely involving the amino nitrogens. HYSCORE spectra of ^15^N‐enriched “TAQ” and TAPT are also very similar (Figures 5E,F and S15). Both spectra exhibit distinct ^15^N features, including weak parallel ridges in the (−,+) quadrant and strong cross‐peaks at approximately (0.67, 2.38), (1.36, 1.59), (1.59, 1.40), and (2.38, 0.67) MHz in the (+,+) quadrant. These patterns are consistent with ^15^N hyperfine interactions that contain a non‐negligible isotropic component and weak‐to‐moderate anisotropic coupling, with a maximum effective coupling of approximately 1.7 MHz, corresponding to approximately 1.2 MHz for ^14^N. This smaller coupling observed in the ^15^N spectra can be attributed to the hyperfine coupling with amino nitrogens. The difference between the dominant ^14^N and ^15^N HYSCORE features suggests that the strongest features in the two spectra do not arise from the same nitrogen site, consistent with the different sensitivities of ^14^N and ^15^N HYSCORE to quadrupolar and hyperfine interactions.

Overall, low‐temperature EPR shows that “TAQ” and TAPT contain both weakly delocalized π‐radicals and a smaller population of coupled spin species, but TAPT favors local spin coupling more strongly. The half‐field measurements reveal minor S ≥ 1, triplet‐like species in both materials, with a much larger and more thermally persistent contribution in TAPT. In contrast, HYSCORE shows that the echo‐detectable S = 1/2 radicals in both “TAQ” and TAPT are weakly delocalized over more than one molecular unit. These results reinforce the picture from temperature‐dependent CW EPR: the more ordered “TAQ” framework supports a more robust delocalized π‐spin manifold, whereas the less ordered TAPT framework promotes localized/weakly delocalized radicals and local spin coupling.

Connecting these results to electronic conductivity, which depends on both charge‐carrier concentration and transport efficiency, we note that TAPT has a room‐temperature radical concentration approximately twice that of “TAQ”, yet “TAQ” exhibits a bulk conductivity that is nearly two orders of magnitude higher. This contrast indicates that the total radical concentration cannot explain the conductivity difference and instead highlights the importance of radical delocalization and structural coherence for efficient charge transport. Thus, the higher conductivity of “TAQ” may be associated with a more ordered hydrogen bonding network and long‐range structural order that support radical delocalization.

Hydrogen bonding may influence electronic transport in “TAQ” in two nonexclusive ways [54]. First, it can serve as a supramolecular organizing interaction that locks neighboring molecular units into a more uniform in‐plane registry, thereby improving crystallinity, reducing structural disorder, and preserving π–π overlap across the layered framework [32, 55, 56, 57, 58]. Second, hydrogen bonding can in some cases be electronically active, because short or highly ordered hydrogen bonds may modulate redox state, electron–proton coupling, radical delocalization, and CT pathways in organic conductors and radical materials [32, 59, 60]. Therefore, while our present data do not prove a direct hydrogen‐bond‐mediated transport pathway in “TAQ”, they show that the resolved hydrogen‐bonding network is associated with longer‐range structural coherence and a more delocalized radical manifold. This relationship provides a plausible structural basis for the higher effective pellet conductivity of “TAQ”, while recognizing that particle morphology, grain boundaries, and other microstructural factors may also influence the absolute conductivity.

## 3 Conclusions

This work shows that “TAQ” and TAPT are not best understood as fundamentally different molecular structures, but as materials with the same molecular building unit organized with different degrees of long‐range order. Although both materials are synthesized from TABQ and show nearly identical short‐range PDF features and matching PXRD peak positions, “TAQ” exhibits sharper diffraction peaks, more persistent long‐range structural correlations, and approximately two orders of magnitude higher bulk electronic conductivity than TAPT.

Solid‐state NMR and MicroED confirm that both materials contain a pyrazine‐based quinonoid structure, while “TAQ” possesses a more ordered hydrogen‐bonding network. ^15^N and ^13^C NMR show no evidence for protonated central nitrogen sites or keto–enol tautomerization. MicroED of “TAQ” independently supports this assignment through bond‐length analysis and experimentally resolved hydrogen positions, which locate protons on the amino nitrogens rather than the central pyrazinic nitrogens. The resolved hydrogen‐bonding network in “TAQ” explains the distinct amine environments observed by ^15^N NMR.

EPR reveals that this structural order controls the distribution of radical species. “TAQ” is dominated by a broad, anisotropic, Dysonian‐like signal consistent with a strongly delocalized π‐spin manifold, whereas TAPT contains a larger fraction of localized or weakly delocalized S = 1/2 radicals. Temperature‐dependent EPR further shows that TAPT undergoes stronger spin localization, trapping, and possible spin‐pairing upon cooling, while the delocalized spin manifold in “TAQ” remains comparatively robust. Low‐temperature half‐field and HYSCORE EPR further show that both materials contain a minor population of coupled S ≥ 1 spin species, together with echo‐detectable S = 1/2 radicals that are weakly delocalized over more than one molecular unit.

In summary, “TAQ” and TAPT provide an example of how synthesis‐dependent differences in long‐range structural order is linked to electronic structure and radical delocalization in organic cathode materials. Both materials share the same molecular structure, but “TAQ” exhibits a higher degree of crystallinity and a more ordered hydrogen‐bonding network. This higher structural order is associated with a more delocalized radical manifold and higher bulk conductivity, whereas the less ordered TAPT structure favors localized and spin‐coupled radical species, resulting in lower bulk conductivity. Because “TAQ” and TAPT also differ in particle morphology, particle size, defect density, and grain‐boundary structure, the present data do not isolate hydrogen‐bond ordering as the sole origin of the conductivity difference. Collectively, the X‐ray scattering, solid‐state NMR, MicroED, and EPR results establish a consistent association among the degree of crystallinity, hydrogen‐bonding order, radical delocalization, and electronic conductivity. Looking forward, controlling crystallinity and hydrogen‐bond topology should provide a practical strategy for tuning radical delocalization and electronic conductivity in redox‐active OEMs.

## Author Contributions

**Alae Eddine Lakraychi**: conceptualization, validation, visualization, investigation, data curation, methodology, writing – original draft, writing – review and editing, formal analysis. **Zeqian Zhang**: investigation, writing – original draft, methodology, formal analysis, data curation, writing – review and editing. **Oliver Lin**: investigation, writing – original draft, writing – review and editing, methodology, data curation, formal analysis. **Eric D Walter**: data curation, methodology, writing – review and editing, resources. **Jiaqi Wang**: methodology, data curation, writing – review and editing. **Harshan Reddy Gopidi**: investigation, methodology, writing – review and editing. **Eliot F. Woods**: investigation, data curation, writing – review and editing. **Ruozhu Feng**: investigation, data curation, funding acquisition, writing – review and editing, resources. **Wei Wang**: investigation, data curation, funding acquisition, writing – review and editing, resources. **Pieremanuele Canepa**: investigation, methodology, funding acquisition, writing – review and editing. **Brett A. Helms**: investigation, funding acquisition, writing – review and editing, conceptualization. **Kamila M. Wiaderek**: investigation, funding acquisition, data curation, methodology, resources, writing – review and editing. **Qian Chen**: investigation, funding acquisition, methodology, data curation, writing – review and editing, resources, validation. **Yan Yao**: conceptualization, investigation, funding acquisition, writing – review and editing, supervision, resources, validation, project administration, visualization, methodology. **Ying Chen**: validation, methodology, conceptualization, investigation, funding acquisition, writing – original draft, writing – review and editing, visualization, formal analysis, data curation, supervision, resources, project administration.

## Conflicts of Interest

Y.Y. has equity interests in LiBeyond, LLC and Solid Design Instruments, LLC. The University of Houston reviewed and approved his relationship in compliance with its conflict‐of‐interest policy. B.A.H. has a financial interest in Sepion Technologies. The remaining authors declare no competing interests.

## Supporting information



Supporting Information contains detailed materials synthesis and characterization methods, crystal structure of “TAQ”, additional solid‐state NMR and EPR spectra. **Supporting File**: anie74832‐sup‐0001‐SuppMat.docx.

## Data Availability

The data that support the findings of this study are available on request from the corresponding author. The data are not publicly available due to privacy or ethical restrictions. Crystallographic data for the structure of “TAQ” is openly available in the Cambridge Crystallographic Data Center (CCDC) (www.ccdc.cam.ac.uk) under the reference no. CCDC 2556563.
