# Supplementary material for: Hydrogen‐Bonding Order Associated With Radical Delocalization in Layered Organic Cathode Materials
Source: Angew Chem Int Ed Engl. 2026 Sep 16;65(45):e2483808. doi: 10.1002/anie.2483808 (PMC13725574; doi:10.1002/anie.2483808)
Supplement: Supplementary file 1 [file ANIE-65-e2483808-s001.docx]

**Hydrogen-Bonding Order Associated with Radical Delocalization in Layered Organic Cathode Materials**

Alae Eddine Lakraychi,^a,b,c,#^ Zeqian Zhang,^a,d,e,#^ Oliver Lin,^f,g^ Eric D Walter,^a,e^ Jiaqi Wang,^a,h^ Harshan Reddy Gopidi, ^a,b,c^ Eliot F. Woods,^a,i^ Ruozhu Feng,^a,i^ Wei Wang,^a,i^ Pieremanuele Canepa,^a,b,c^ Brett A. Helms,^a,j,k^ Kamila M. Wiaderek,^a,h^ Qian Chen,^f,g,l^ Yan Yao,^a,b,c,m,n*^ and Ying Chen^a,e*^

^a^Energy Storage Research Alliance, Argonne National Laboratory, 9700 South Cass Avenue, Lemont, IL 60439, USA

^b^Department of Electrical and Computer Engineering, University of Houston, Houston, TX 77204, USA

^c^Texas Center for Superconductivity, University of Houston, Houston, TX 77204, USA

^d^Department of Chemistry, University of Washington, Seattle, WA 98195, USA

^e^Physical and Computational Sciences Directorate, Paciﬁc Northwest National Laboratory, Richland, WA 99352, USA

^f^Department of Chemistry, University of Illinois, Urbana, IL 61801, USA

^g^Materials Research Laboratory, University of Illinois, Urbana, IL 61801, USA.

^h^X-ray Science Division, Argonne National Laboratory, Lemont, IL 60439, USA

^i^Energy and Environment Directorate, Pacific Northwest National Laboratory, Richland, WA 99354, USA

^j^The Molecular Foundry, Lawrence Berkeley National Laboratory, Berkeley, CA 94720, USA

^k^Materials Sciences Division, Lawrence Berkeley National Laboratory, Berkeley, CA 94720, USA

^l^Department of Materials Science and Engineering, University of Illinois, Urbana, IL 61801, USA.

^m^Department of Earth and Environmental Engineering, Columbia University, New York, NY 10027

^n^Columbia Climate School, Columbia University, New York, NY 10027

^#^These authors contributed equally to this work

*E-mail: [yy3784@columbia.edu](mailto:yy3784@columbia.edu) *E-mail: [chen675@pnnl.gov](mailto:chen675@pnnl.gov)

**Contents**

[**Materials.**	3](#_Toc237201770)

[**Materials synthesis.**	3](#_Toc237201771)

[**Characterization Methods**	5](#_Toc237201772)

[*Fourier transform infrared (FTIR) and scanning electron microscopy (SEM)*	5](#_Toc237201773)

[*Synchrotron X-ray diffraction (SXRD) and pair distribution function (PDF)*	5](#_Toc237201774)

[*Diffuse Reflectance Ultraviolet-Visible-Near Infrared (DRUV-Vis-NIR)*	5](#_Toc237201775)

[*Solid-state nuclear magnetic resonance (NMR)*	6](#_Toc237201776)

[*Electron paramagnetic resonance (EPR)*	6](#_Toc237201777)

[*Electronic conductivity measurements*	7](#_Toc237201778)

[*Microcrystal electron diffraction (MicroED)*	8](#_Toc237201779)

[**Characterization of “TAQ” and TAPT**	10](#_Toc237201780)

[**Solid-state NMR spectra**	11](#_Toc237201781)

[**Crystal structure of “TAQ”**	16](#_Toc237201782)

[**Characterization of TAPT***	20](#_Toc237201783)

[**EPR characterization of “TAQ” and TAPT**	22](#_Toc237201784)

[**References**	26](#_Toc237201785)

# **Materials.**

The following chemicals and solvents were purchased from commercial suppliers and used without further purification. Tetraaminobenzoquinone (TABQ) was synthesized following a previous report.^1^ 2,3,5,6–Tetrachloro–1,4-benzoquinone (chloranil, TCI Chemical), phthalimide potassium salt (98%, Thermo Chemical), ^15^N-phthalimide potassium salt (98%, Cambridge Isotope Laboratories), hydrazine monohydrate (98%, TCI Chemical), tetrabutylammonium chloride (TABCl 97%, Sigma-Aldrich), sodium acetate (99%, Sigma-Aldrich), dimethylformamide (DMF, HPLC grade, Thermo Chemical), acetonitrile (HPLC grade, Thermo Chemical), hydrochloric acid (37%, Sigma-Aldrich).

# **Materials synthesis.**

*Bis-tetraaminobenzoquinone (“TAQ”) synthesis*: “TAQ” was synthesized by self-condensation of the precursor TABQ, following an adapted procedure based on previous reports.^1–5^ 250 mL of dimethylformamide was added to a 500 mL Schlenk flask, followed by addition of 37.6 g of TABCl. The mixture was sonicated until a clear and colorless solution was obtained. Subsequently, 4.0 g of TABQ was added, resulting in a dark green solution, which was further sonicated for an additional 10 minutes before being heated in an oil bath at 85 °C overnight. After cooling to room temperature, the resulting dark mixture was filtered, and the isolated solid was thoroughly washed with dimethylformamide and methanol. The final dark greenish black solid was dried under vacuum at 80 °C overnight, affording “TAQ” in 35% yield. For the synthesis of ^15^N-”TAQ”, ^15^N-TABQ was first prepared using ^15^N-phthalimide potassium salt according to the reported procedure.^1^ Subsequent self-condensation of ^15^N-TABQ was conducted following the procedure described above to afford the product with 30% yield.

IR (“TAQ”) 𝜐̃ = 3464, 3403, 3341, 3287, 3228, 1651, 1620, 1518, 1461, 1345, 1234, 1184, 1130, 1026, 718, 656, 585 cm^−1^.

*2,3,7,8-tetraaminophenazine-1,4,6,9-tetraone (TAPT) synthesis:* TAPT was synthesized by self-condensation of the precursor TABQ according to the previously reported aqueous method.^6^ 4.0 g TABQ was dispersed in 250 mL of deionized water containing 4.0 ml of hydrochloric acid and sonicated for 30 min to obtain a dark red suspension. Subsequently, 25.9 g of sodium acetate was added, resulting in the formation of a back mixture. The reaction mixture was then heated to 85 °C and maintained under reflux for 12 h under a continuous flow of O_2_. After completion, the mixture was filtered and thoroughly washed with deionized water. The resulting dark black solid was dried under vacuum at 80 °C overnight, affording TAPT in 80% yield. ^15^N-TAPT was synthesized using the same procedure from ^15^N-”TAQ”B, affording the product with 71% yield.

2,3,7,8-tetraaminophenazine-1,4,6,9-tetraone (TAPT*) synthesis: TAPT* was synthesized according to the above procedure with a slight modification. Briefly, 4.0 g of TABQ and stir bar were charged into a flask. A solution of 15.3 g of sodium acetate in 200 mL of deionized water was made and the pH adjusted with a slow addition of 8.7 mL concentrated HCl acid. The acidic sodium acetate solution is then added to the TABQ, and the reaction was set to stir vigorously at 100 °C under a stream of compressed air for 3 h. The temperature of the reaction was then lowered to 80 °C and the stream of compressed air removed. The reaction was stirred open to air at 80 °C for 12 h before being cooled to room temperature and filtered. The filter cake was washed with 50 mL of DMF and a large excess of water to yield TAPT* as a black solid in 62% yield.

IR (TAPT) 𝜐̃ = 3439, 3445, 3279, 3219, 3159, 1651, 1620, 1518, 1461, 1345, 1234, 1184, 1130, 1026, 718, 656, 585 cm^−1^.

# **Characterization Methods**

## *Fourier transform infrared (FTIR) and scanning electron microscopy (SEM)*

Infrared spectra were measured in attenuated total reflectance (ATR) mode on a Nicolet iS5 spectrometer (Thermo Scientific) in the range of 600-4000 cm^–1^ at a resolution of 2 cm^–1^ with 64 scans.

SEM imaging was performed Axia ChemiSEM operated at accelerating voltages of 5 kV. “TAQ” and TAPT powders were coated with a thin layer of gold prior to imaging.

## *Synchrotron X-ray diffraction (SXRD) and pair distribution function (PDF)*

SXRD and PDF experiments were performed at the 11-ID-B beamline of the Advanced Photon Source, Argonne National Laboratory, using an incident wavelength of 0.2099 Å. CeO₂ was employed as the calibration standard. The powders were loaded into Kapton capillaries and sealed at both ends with clay. The one-dimensional (1D) scattering profiles were obtained by integrating the two-dimensional (2D) scattering data using GSAS-II.^7^ The corresponding PDFs were extracted with xPDFsuite.

## *Diffuse Reflectance Ultraviolet-Visible-Near Infrared (DRUV-Vis-NIR)*

*DRUV-Vis-NIR spectra were collected using a JASCO ArMN-9211 spectrophotometer over the wavelength range of 200–1600 nm at a scan rate of 1000 nm min^–1^ under ambient conditions. The measurements were performed using a 90° incidence geometry. Approximately 100 mg of each powder was ground using a mortar and pestle, transferred into a custom-made cylindrical polyetheretherketone (PEEK) die with an inner diameter of 13 mm, and cold-pressed at 3 tons for 3 min to form a dense pellet.*

## *Solid-state nuclear magnetic resonance (NMR)*

^1^H, ^13^C and ^15^N magic angle spinning (MAS) NMR spectra were collected on a Bruker Avance III spectrometer with a field strength of 600 MHz (14.1 T) using 3.2 mm NMR rotors at a spinning speed of 10-20 kHz. The 90˚ pulse width was 3.2 µs for ^1^H, 3.3 µs for ^13^C, and 3.3 µs for ^15^N. The ^1^H and ^15^N direct polarization (DP) spectra were obtained using single pulse excitation with 128 scans and a recycle delay of 1200 s for quantitative comparison. ^15^N spin-lattice relaxation time (*T*_1_) measurements utilized the saturation-recovery method. ^1^H-^13^C and ^1^H-^15^N cross polarization (CP) and ^1^H-^15^N HETCOR were acquired using the pulse sequence “cp” and “lghetfq” at Topspin, with the CP contact time varying from 50 µs to 3000 µs. The LG-RF field was set at 100 kHz and spinning speed of 12 kHz for HETCOR. The ^1^H, ^13^C, and ^15^N chemical shifts were externally referenced to adamantane at 1.91 ppm, adamantane at 38.5/29.5 ppm, and ^15^N-enriched γ-glycine at 33.6 ppm, respectively.

## *Electron paramagnetic resonance (EPR)*

Electron paramagnetic resonance (EPR) experiments were carried out on a Bruker E580 X-band spectrometer. All samples were packed in 4-mm quartz EPR tubes sealed by Critoseal under a N_2_ glove box. For continuous-wave (CW) experiments, the spectrometer was equipped with a SHQE resonator and a continuous flow cryostat. The typical settings for acquiring the spectra were microwave frequency = 9.373 GHz, sweep width = 100 G, sweep time = 20.97 s, power = 0.0002 mW, and field modulation amplitude = 0.25 G. The settings for half-field EPR were sweep width = 5000 G (perpendicular) or 4000 G (parallel), sweep time = 41.94 s, power = 20 mW, and field modulation amplitude = 10 G.

For pulsed EPR experiments the spectrometer was outfitted with an MD-5 dielectric resonator and an Oxford liquid helium continuous flow cryostat which maintained the temperature at 5 K. Typical parameters for HYSCORE experiments with a pi/2-tau-pi/2-t1-pi-t2-pi/2 sequence were: pi/2 = 8 ns, pi = 16 ns, tau = 128, 180, and 220 ns, and t1, t2 ranging from 200 ns to 4296 ns in 256 steps of 16 ns. The recycle delay was ~ 50 ms and typically 4 shots were collected for each delay with phase cycling.

EPR fittings were performed using the Pepper function in EasySpin.^8^ All simulations were initially conducted using a single-component model with three g values set close to 2.002 and an initial mwPhase value at 0. The mwPhase parameter was set within the range of 0 to 0.57. A second component was introduced only when the single-component model failed to provide a satisfactory fit within the defined mwPhase range.

## *Electronic conductivity measurements*

The electronic conductivities of “TAQ” and TAPT were measured by DC polarization in an ion-blocking cell with a four-probe configuration.^9^ Briefly, 100 mg of sample powders were placed inside a cylindrical custom-made PEEK die cell with an inner diameter of 13 mm and then cold-pressed at 3 tons for 3 min to form a dense pellet. Next, 10 mg of carbon black (Csp) were uniformly distributed on both sides of the pellet, followed by an additional cold-pressing step at 3 tons for 2 min. The resulting three-layer Csp│sample│Csp cell was assembled and tightened under an external pressure of 10 MPa. Measurements were carried out by applying stepwise voltages, with each voltage step maintained for 20 minutes to record the steady-state current response, using a Bio-logic SP-50 galvanostat/potentiostat. The applied voltages were plotted against the corresponding currents (V-I curves) to extract the pellet resistance (R) as the slope of the linear fit. The measurements were conducted at 20 °C. The electronic conductivity was calculated based on the equation 𝜎=L/(R S), where L is the thickness of the pellet, S is the area of the surface, and R is the resistance.

## *Microcrystal electron diffraction (MicroED)*

Microcrystalline “TAQ” powder was deposited directly on Quantifoil Cu 200 R 2/2 holely carbon TEM grid (4430C-CF, SPI) inside an argon glovebox without additional surface treatment. Upon removal from the inert environment, the grids were immediately immersed into liquid nitrogen and stayed in liquid nitrogen environment until loaded into an autoloader and then a cryogenic electron microscope. MicroED data collection was performed on a ThermoFisher Glacios operating at 200 kV at 100 K. Data were acquired using built-in software EPU-D for continuous-rotation method, in which sample stage was continuously rotating around a single axis with diffraction pattern being continuously recorded on a charge-coupled device (CCD) camera, Ceta-D (2048 × 2048 pixels). Parallel illumination condition was used at spot size 10, a 20 μm condenser aperture, and a calibrated camera length of 0.67 m (corresponding to 0.58 Å at the detector edge). The rotation rate was 1°/s with exposure time of 1 s. Microcrystal 1 and Microcrystal 2 covered rotation range of –55° to 55° and –60° to 60° respectively.

Initial indexing, integration, and scaling of the two datasets were performed individually in DIALS (v3.14) to assess completeness, signal-to-noise ratio ($I/\sigma)$, and inter-dataset consistency $(R_{merge})$. For datasets exhibiting suitable statistics, the corresponding frames were imported into PETS2 to determine averaged beam-center coordinates, which were subsequently supplied to DIALS for a second round of processing. In this second pass, frame ranges were reevaluated and non-optimal frames were removed to maintain low merging statistics and stable refinement of the orientation matrix. For Microcrystal 1, all 110 frames were retained, yielding $92\%$ completeness. For Microcrystal 2, 120 frames were used after excluding frames 35, 47, 50, 62, 79, 110, 111, and 118–120, resulting $99\%$ completeness, $R_{int}$ of $9.00\%$, and $I/\sigma$ of 18.1. The two optimized datasets were then re-integrated up to 0.65 Å and merged in DIALS to achieve 100% completeness. The merged reflection file was subsequently imported to XPREP and Olex2 for structural solution and refinement. The atomic displacement parameters (ADPs) for the non-hydrogen atoms were refined anisotropically, while the hydrogen atoms were isotropically and freely. More refinement details can be found in Table S2–6 and CIF file. The final MicroED structure refined to $R_{1}=15.12\%$, ${wR}_{2}=34.95\%$, and Goodness-of-fit of $1.239$.

# **Characterization of “TAQ” and TAPT**

**Figure S1.** Overlay of PXRD patterns of multiple batches of “TAQ”, highlighting the reproducibility of both syntheses.

**Figure S2.** FTIR spectra of “TAQ” and TAPT.

**Figure S3.** Overlay of the DRUV-Vis-NIR spectra of “TAQ” and TAPT.

# **Solid-state NMR spectra**

**Figure S4.** Quantitative ^15^N DP NMR of TAPT and “TAQ” showing the molar ratio between amino N and pyrazinic N is 2:1. Spinning sidebands associated with the pyrazinic N resonance are indicated by asterisks.

**Figure S5.** ^1^H-^15^N CP spectrum at long CP contact time (3000 µs) and large spectral window showing no piperazine or imine-type of N sites. DDDPD refers to 2,7-diamino-3,8-diimino-1,6-dihydroxyphenazine-4,9-dione.

**Figure S6.** ^15^N NMR signal attenuation during saturation-recovery measurements for the (A) pyrazinic and (B) amino N of “TAQ”.

**Figure S7.** ^1^H–^13^C CP spectra acquired at varying CP contact times for reassignment of the 145 ppm resonance.

If keto–enol tautomerization were present, the –C=NH signal at 145 ppm should exhibit CP efficiency similar to that of the C=C–NH_2_ signals at 130–135 ppm. Instead, it shows CP behavior similar to that of the C=O signal at 175 ppm, supporting reassignment of the 145 ppm resonance to the four carbon atoms bonded to the pyrazinic N atoms.

**Table S1.** Comparison of ^13^C chemical shifts (ppm) for the three carbon environments C=O, C-NH_2_, and N=C-C in “TAQ” and TAPT, including previously reported values from literature reports,^1,6^ our experimental measurements, and empirical prediciton^10^ for “TAQ” with a protonated piperazine-like center and TAPT with a deprotonated purazine-like center. For comparison of the relative chemical-shift patterns, the predicted shifts were referenced by setting the C=O resonance to 175.0 ppm. Although intermolecular hydrogen bonding in the crystal structure can influence ^13^C chemical shifts, the predicted values for molecular TAPT show overall better agreement with the experimental data than those for “TAQ”. The ChemInfo predictor^10^ is based on experimental database entries and HOSE-code molecular environments and does not explicitly account for crystal packing, intermolecular hydrogen bonding, π–π stacking, or other solid-state effects. The predicted molecular TAPT shifts show better overall agreement with the experimental values, with deviations of less than 7 ppm, whereas the molecular “TAQ” model gives deviations greater than 15 ppm for the C–NH_2_ and N=C–C environments. These results support protonation states and covalent connectivities for both “TAQ” and TAPT that are more consistent with a deprotonated pyrazine center. The remaining differences between the molecular predictions and experimental values may reflect solid-state intermolecular interactions and the intrinsic limitations of the empirical prediction method.

| Material | C=O | C-NH_2_ | N=C-C | |
| --- | --- | --- | --- | --- |
| “TAQ” from Ref ^1^ | 169.27 | 140.83 | 129.06 | 124.53 |
| “TAQ” from this work | 174.2 | 145.9 | 134.0 | 129.4 |
| Difference | 4.93 | 5.07 | 4.94 | 4.87 |
| TAPT from Ref ^6^ | 174.2 | 145.35 | 131.54 | |
| TAPT from this work | 174.6 | 146.0 | 132.0 | |
| Difference | 0.4 | 0.65 | 0.46 | |
| Predicted TAQ | 175.0 | 129.9 | 112.2 | |
| Predicted TAPT | 175.0 | 141.6 | 138.7 | |

# **Crystal structure of “TAQ”**

**Figure S8**. (A) Crystal structure of “TAQ” and the packing viewed along the (B) a-axis, (C) b-axis, and (D) c-axis. Hydrogen bonds are shown as dashed lines. Non-hydrogen atoms are represented as thermal ellipsoids at the 50% probability level.

**Table S2.** Crystal data and structure refinement for “TAQ”

| Empirical formula | C_12_H_8_N_6_O_4_ |
| --- | --- |
| Formula weight | 300.23 |
| Temperature (K) | 100 |
| Crystal system | monoclinic |
| Space group (number) | $P2_{1}/c$ (14) |
| *a, b, c* (Å) | 4.8936(5), 11.285(1), 9.841(1) |
| α, β, γ (°) | 90, 102.097(9), 90 |
| Volume (Å^3^) | 531.37(10) |
| *Z* | 2 |
| *ρ*_calc_ (gcm^−3^) | 1.876 |
| Crystal color, shape | Black, Rod |
| Radiation | Electron (200kV, λ=0.02508 Å) |
| No. of crystals | 2 |
| 2θ range (°) | 0.20 to 2.21 (0.65 Å) |
| Index ranges | −7 ≤ h ≤ 7 −17 ≤ k ≤ 17 −15 ≤ l ≤ 15 |
| Reflections collected | 8663 |
| Independent reflections | 2031  *R*_int_ = 0.0900 *R*_sigma_ = 0.0553 |
| Completeness to  θ = 0.862° | 100.0 % |
| Data / Restraints / Parameters | 2031 / 37 / 117 |
| Goodness-of-fit on *F*^2^ | 1.259 |
| Final *R* indexes  (*I*≥2σ(*I*)) | *R*_1_ = 0.1503 w*R*_2_ = 0.3456 |
| Largest peak/hole (eÅ^−3^) | 0.21, −0.16 |

**Table S3.** Bond lengths for “TAQ”

| **Atom** | **Atom** | **Length/Å** |  | **Atom** | **Atom** | **Length/Å** |
| --- | --- | --- | --- | --- | --- | --- |
| O1 | C2 | 1.362(7) |  | O1A | C2A | 1.359(10) |
| O2 | C3 | 1.327(9) |  | O2A | C3A | 1.350(10) |
| C1 | C2 | 1.429(9) |  | C1A | C2A | 1.425(10) |
| C1 | C7^1^ | 1.404(10) |  | C1A | C7A^2^ | 1.389(8) |
| C1 | C8^1^ | 1.411(8) |  | C1A | C8A^2^ | 1.415(10) |
| C2 | C3 | 1.374(11) |  | C2A | C3A | 1.371(9) |
| C3 | C4 | 1.438(8) |  | C3A | C4A | 1.429(10) |
| C4 | C5 | 1.391(11) |  | C4A | C5A | 1.396(8) |
| C4 | C8 | 1.415(8) |  | C4A | C8A | 1.409(10) |
| C5 | C6 | 1.383(10) |  | C5A | C6A | 1.359(10) |
| C6 | C7 | 1.381(10) |  | C6A | C7A | 1.389(11) |
| C8 | C8^1^ | 1.426(14) |  | C8A | C8A^2^ | 1.435(10) |

*¹ 1−X, 1−Y, 2−Z; ² −X, 2−Y, 1−Z*

**Table S4.** Bond angles for “TAQ”

| **Atom** | **Atom** | **Atom** | **Angle/°** |  | **Atom** | **Atom** | **Atom** | **Angle/°** |
| --- | --- | --- | --- | --- | --- | --- | --- | --- |
| C7^1^ | C1 | C2 | 122.4(5) |  | C7A^2^ | C1A | C2A | 123.2(7) |
| C7^1^ | C1 | C8^1^ | 118.9(6) |  | C7A^2^ | C1A | C8A^2^ | 118.5(7) |
| C8^1^ | C1 | C2 | 118.6(7) |  | C8A^2^ | C1A | C2A | 118.3(5) |
| O1 | C2 | C1 | 121.1(7) |  | O1A | C2A | C1A | 122.3(6) |
| O1 | C2 | C3 | 117.1(6) |  | O1A | C2A | C3A | 116.2(7) |
| C3 | C2 | C1 | 121.6(5) |  | C3A | C2A | C1A | 121.5(7) |
| O2 | C3 | C2 | 121.5(6) |  | O2A | C3A | C2A | 120.9(7) |
| O2 | C3 | C4 | 118.1(7) |  | O2A | C3A | C4A | 118.3(6) |
| C2 | C3 | C4 | 120.3(6) |  | C2A | C3A | C4A | 120.7(7) |
| C5 | C4 | C3 | 121.0(6) |  | C5A | C4A | C3A | 121.1(7) |
| C5 | C4 | C8 | 119.9(6) |  | C5A | C4A | C8A | 119.5(7) |
| C8 | C4 | C3 | 119.0(6) |  | C8A | C4A | C3A | 119.4(5) |
| C6 | C5 | C4 | 120.0(7) |  | C6A | C5A | C4A | 120.0(7) |
| C7 | C6 | C5 | 120.9(8) |  | C5A | C6A | C7A | 121.4(6) |
| C6 | C7 | C1^1^ | 120.6(6) |  | C1A^2^ | C7A | C6A | 120.6(7) |
| C1^1^ | C8 | C4 | 119.6(6) |  | C1A^2^ | C8A | C8A^2^ | 120.6(8) |
| C1^1^ | C8 | C8^1^ | 120.4(7) |  | C4A | C8A | C1A^2^ | 120.0(5) |
| C4 | C8 | C8^1^ | 120.0(6) |  | C4A | C8A | C8A^2^ | 119.4(8) |

*¹ 1−X, 1−Y, 2−Z; ² −X, 2−Y, 1−Z*

**Table S5.** Hydrogen bonds for “TAQ”

| **D** | **H** | **A** | **d(D−H)/Å** | **d(H−A)/Å** | **d(D−A)/Å** | **D−H−A/°** |
| --- | --- | --- | --- | --- | --- | --- |
| O1 | H1 | O1A | 0.91(4) | 2.12(4) | 2.834(8) | 134(4) |
| O2 | H2 | O2A^1^ | 0.98(4) | 1.97(4) | 2.894(8) | 157(5) |
| O1A | H1A | O1^2^ | 0.97(4) | 1.80(4) | 2.678(8) | 150(4) |
| O2A | H2A | O1A | 0.89(5) | 2.20(4) | 2.665(8) | 112(4) |

*¹ 1−X, 1−Y, 1−Z; ² 1+X, +Y, +Z*

**Table S6.** Torsion angles for “TAQ”

| **A** | **B** | **C** | **D** | **Angle/°** |  | **A** | **B** | **C** | **D** | **Angle/°** |
| --- | --- | --- | --- | --- | --- | --- | --- | --- | --- | --- |
| O1 | C2 | C3 | O2 | 0.3(10) |  | O1A | C2A | C3A | O2A | 3.2(8) |
| O1 | C2 | C3 | C4 | 176.9(6) |  | O1A | C2A | C3A | C4A | 179.8(5) |
| O2 | C3 | C4 | C5 | −5.1(11) |  | O2A | C3A | C4A | C5A | −3.6(8) |
| O2 | C3 | C4 | C8 | 177.0(6) |  | O2A | C3A | C4A | C8A | 178.3(5) |
| C1 | C2 | C3 | O2 | −175.0(7) |  | C1A | C2A | C3A | O2A | −179.1(5) |
| C1 | C2 | C3 | C4 | 1.7(11) |  | C1A | C2A | C3A | C4A | −2.5(8) |
| C2 | C3 | C4 | C5 | 178.1(7) |  | C2A | C3A | C4A | C5A | 179.7(5) |
| C2 | C3 | C4 | C8 | 0.2(11) |  | C2A | C3A | C4A | C8A | 1.6(8) |
| C3 | C4 | C5 | C6 | −176.9(8) |  | C3A | C4A | C5A | C6A | 179.8(5) |
| C3 | C4 | C8 | C1^1^ | 179.2(6) |  | C3A | C4A | C8A | C1A^2^ | 179.7(5) |
| C3 | C4 | C8 | C8^1^ | −1.3(12) |  | C3A | C4A | C8A | C8A^2^ | −0.6(9) |
| C4 | C5 | C6 | C7 | −2.9(13) |  | C4A | C5A | C6A | C7A | 0.9(9) |
| C5 | C4 | C8 | C1^1^ | 1.2(11) |  | C5A | C4A | C8A | C1A^2^ | 1.6(8) |
| C5 | C4 | C8 | C8^1^ | −179.2(8) |  | C5A | C4A | C8A | C8A^2^ | −178.7(6) |
| C5 | C6 | C7 | C1^1^ | 2.4(12) |  | C5A | C6A | C7A | C1A^2^ | 0.8(9) |
| C7^1^ | C1 | C2 | O1 | 2.6(10) |  | C7A^2^ | C1A | C2A | O1A | −0.9(8) |
| C7^1^ | C1 | C2 | C3 | 177.7(7) |  | C7A^2^ | C1A | C2A | C3A | −178.5(6) |
| C8^1^ | C1 | C2 | O1 | −177.5(6) |  | C8A^2^ | C1A | C2A | O1A | 179.8(5) |
| C8^1^ | C1 | C2 | C3 | −2.5(10) |  | C8A^2^ | C1A | C2A | C3A | 2.2(8) |
| C8 | C4 | C5 | C6 | 1.0(12) |  | C8A | C4A | C5A | C6A | −2.1(9) |

*¹ 1−X, 1−Y, 2−Z; ² −X, 2−Y, 1−Z*

# **Characterization of TAPT***

**Figure S9.** Powder X-ray diffraction pattern (PXRD) of TAPT* and TAPT.

Both TAPT and TAPT* were synthesized from the same starting materials—TABQ, hydrochloric acid, and sodium acetate—in deionized water under an oxygen-containing atmosphere. The key difference lies in the reaction conditions: TAPT was synthesized at 85 °C under continuous O_2_ flow for 12 h, whereas TAPT* was prepared at 100 °C under compressed air with vigorous stirring for 3 h, as detailed in the Materials Synthesis section. Both routes yield the same molecular structure and materials that are less crystalline than “TAQ”. However, the higher temperature and vigorous stirring used for TAPT* result in even lower crystallinity than TAPT, as evidenced by the broader PXRD reflections (Figure S9). The peak at 2θ = 27.43° indicates that TAPT* exhibits an interlayer distance similar to that of TAPT (≈ 3.2 Å).

**Figure S10.** Two-component fits of the EPR spectra of TAPT*: (A) X-band CW EPR spectrum collected at 293 K, (B) Q-band spectrum collected at 293 K, and (C) Q-band spectrum collected at 5 K. Black circles represent the experimental data, while the green, blue, and red lines correspond to the broad component, sharp component, and summed fit, respectively.

# **EPR characterization of “TAQ” and TAPT**

**Figure S11.** CW EPR spectra and corresponding fittings at varying temperatures for “TAQ”.

**Figure S12.** CW EPR spectra and corresponding fittings at varying temperatures for TAPT.

**Figure S13.** Temperature dependence of mwPhase for “TAQ” and TAPT.

**Figure S14.** Temperature dependence of mass-normalized DI for “TAQ” and TAPT.

**Figure S15.** Temperature dependence of g-tensor for TAPT.

**Figure S16.** EPR signal attenuation for phase-memory time measurements for natural abundant “TAQ” vs TAPT.

**Figure S17.** HYSCORE of natural abundant and ^15^N-enriched “TAQ” and TAPT at varying inter-pulse delays (128 ns, 180 ns, and 220 ns) to avoid blind spots.

# **References**

(1)	Chen, T.; Banda, H.; Yang, L.; Li, J.; Zhang, Y.; Parenti, R.; Dincă, M. High-Rate, High-Capacity Electrochemical Energy Storage in Hydrogen-Bonded Fused Aromatics. *Joule* **2023**, *7* (5), 986–1002. https://doi.org/10.1016/j.joule.2023.03.011.

(2)	Chen, T.; Banda, H.; Wang, J.; Oppenheim, J. J.; Franceschi, A.; Dincǎ, M. A Layered Organic Cathode for High-Energy, Fast-Charging, and Long-Lasting Li-Ion Batteries. *ACS Cent. Sci.* **2024**, *10* (3), 569–578. https://doi.org/10.1021/acscentsci.3c01478.

(3)	Chen, T.; Wang, J.; Tan, B.; Zhang, K. J.; Banda, H.; Zhang, Y.; Kim, D.-H.; Dincă, M. High-Energy, High-Power Sodium-Ion Batteries from a Layered Organic Cathode. *J. Am. Chem. Soc.* **2025**, *147* (7), 6181–6192. https://doi.org/10.1021/jacs.4c17713.

(4)	Tan, B.; Chen, T.; Wang, J.; Dincă, M. A Metal-Free Cathode for High-Performance Potassium-Ion Batteries under Ambient and Sub-Zero Temperatures. *Watt* **2026**, *1* (1), 5. https://doi.org/10.1007/s44503-026-00005-1.

(5)	Mo, J.; Wang, J.; Dincă, M. Single-Crystalline, Semiconductive Layered Organic Cathode Powers High-Energy All-Solid-State Batteries. *ACS Cent. Sci.* **2026**. https://doi.org/10.1021/acscentsci.6c00267.

(6)	Li, Z.; Jia, Q.; Chen, Y.; Fan, K.; Zhang, C.; Zhang, G.; Xu, M.; Mao, M.; Ma, J.; Hu, W.; Wang, C. A Small Molecular Symmetric All-Organic Lithium-Ion Battery. *Angewandte Chemie International Edition* **2022**, *61* (33), e202207221. https://doi.org/10.1002/anie.202207221.

(7)	Toby, B. H.; Von Dreele, R. B. GSAS-II: The Genesis of a Modern Open-Source All Purpose Crystallography Software Package. *Journal of Applied Crystallography* **2013**, *46* (2), 544–549.

(8)	Stoll, S.; Schweiger, A. EasySpin, a Comprehensive Software Package for Spectral Simulation and Analysis in EPR. *Journal of Magnetic Resonance* **2006**, *178* (1), 42–55. https://doi.org/10.1016/j.jmr.2005.08.013.

(9)	Amin, R.; Chiang, Y.-M. Characterization of Electronic and Ionic Transport in Li1-xNi0.33Mn0.33Co0.33O2 (NMC333) and Li1-xNi0.50Mn0.20Co0.30O2 (NMC523) as a Function of Li Content. *Journal of The Electrochemical Society* **2016**, *163* (8), A1512. https://doi.org/10.1149/2.0131608jes.

(10)	Https://Www.Cheminfo.Org/Spectra/NMR/Predictions/13C_Prediction/Index.Html.
